# The confound of hemodynamic response function variability in human resting-state functional MRI studies

**DOI:** 10.3389/fnins.2023.934138

**Published:** 2023-07-14

**Authors:** D. Rangaprakash, Robert L. Barry, Gopikrishna Deshpande

**Affiliations:** ^1^Athinoula A. Martinos Center for Biomedical Imaging, Massachusetts General Hospital, Harvard Medical School, Charlestown, MA, United States; ^2^Department of Radiology, Harvard Medical School, Boston, MA, United States; ^3^Harvard-Massachusetts Institute of Technology Division of Health Sciences and Technology, Cambridge, MA, United States; ^4^Department of Electrical and Computer Engineering, AU MRI Research Center, Auburn University, Auburn, AL, United States; ^5^Department of Psychological Sciences, Auburn University, Auburn, AL, United States; ^6^Center for Neuroscience, Auburn University, Auburn, AL, United States; ^7^Alabama Advanced Imaging Consortium, Birmingham, AL, United States; ^8^Key Laboratory for Learning and Cognition, School of Psychology, Capital Normal University, Beijing, China; ^9^Department of Psychiatry, National Institute of Mental Health and Neurosciences, Bangalore, India; ^10^Centre for Brain Research, Indian Institute of Science, Bangalore, India

**Keywords:** BOLD fMRI, HRF, resting state connectivity (rsfMRI), aging, sex differences, confound

## Abstract

Functional magnetic resonance imaging (fMRI) is an indirect measure of neural activity with the hemodynamic response function (HRF) coupling it with unmeasured neural activity. The HRF, modulated by several non-neural factors, is variable across brain regions, individuals and populations. Yet, a majority of human resting-state fMRI connectivity studies continue to assume a non-variable HRF. In this article, with supportive prior evidence, we argue that HRF variability cannot be ignored as it substantially confounds within-subject connectivity estimates and between-subjects connectivity group differences. We also discuss its clinical relevance with connectivity impairments confounded by HRF aberrations in several disorders. We present limited data on HRF differences between women and men, which resulted in a 15.4% median error in functional connectivity estimates in a group-level comparison. We also discuss the implications of HRF variability for fMRI studies in the spinal cord. There is a need for more dialogue within the community on the HRF confound, and we hope that our article is a catalyst in the process.

## 1. Introduction

**Figure 1:**
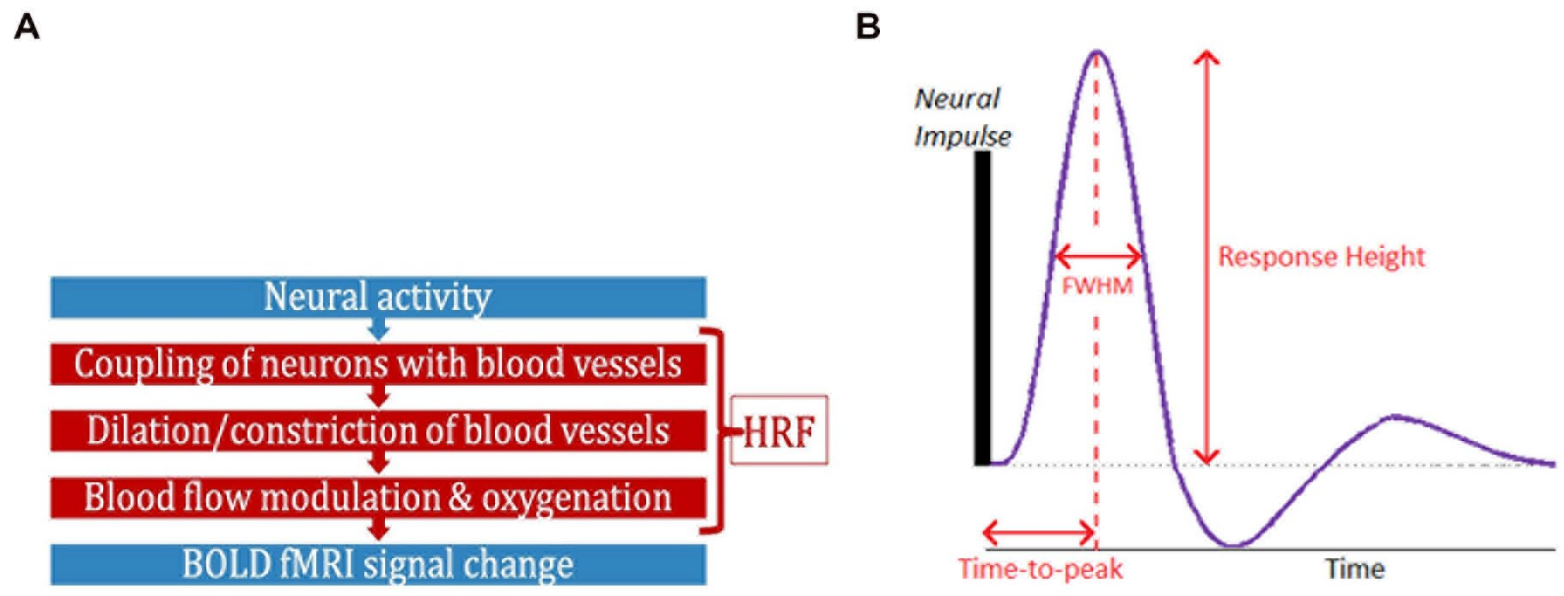
**(A)** FMRI is an indirect measure of neural activity. What stands between them is the hemodynamic response function (HRF). **(B)** The HRF is the BOLD response to a neural impulse. It has a peak (response height) occurring sometime after the neural impulse (time-to-peak) (HRF width = FWHM). The HRF always has this shape (biological), but these parameters are variable.

Functional magnetic resonance imaging (fMRI) has contributed significantly to the advancement of neuroscience, psychiatry, and neurology over the past three decades (Power et al., 2011; Rosazza and Minati, 2011; Nathan et al., 2014). While neural activity can be directly measured *in vivo* through invasive procedures, blood oxygenation level-dependent (BOLD) fMRI is a complex, indirect measure of neural activity (Logothetis et al., 2001; Figure 1A), measuring local blood oxygenation variations in response to active neurons. Dilation and constriction of blood vessels modulates this process, which, in turn, is modulated through numerous non-neural and neural factors that are difficult to delineate (Biessmann et al., 2012). The combination of factors that lie between neural activity and BOLD is the hemodynamic response function (HRF; Duarte et al., 2015; Kim and Ress, 2016). HRF shape is characterized by its amplitude (response height, RH), latency (time-to-peak, TTP) and width (full-width at half max, FWHM; Figure 1B). Representing neurovascular coupling in the BOLD signal, the HRF is modulated by several non-neural factors (Biessmann et al., 2012; Yang et al., 2019) such as hematocrit, variable size/density of vasculature, global magnetic susceptibilities, alcohol/caffeine/lipid ingestion, pulse/respiration differences, and partial volume imaging of veins (Aguirre et al., 1998; Levin et al., 1998; Noseworthy et al., 2003; Handwerker et al., 2004; Buxton, 2010; Tong and Frederick, 2014; Bernier et al., 2018; Yang et al., 2019). The HRF shape varies across the brain and individuals (Aguirre et al., 1998; Handwerker et al., 2004; Lewis et al., 2018).

The current ‘perspective’ article focuses on this HRF variability (HRFv) and its impact on fMRI data processing and subsequent outcome measures within the scope of human fMRI research. This topic is important because thousands of human fMRI studies are published each year, but a significant portion of those do not account for HRFv. Resting-state fMRI (rs-fMRI) and connectivity studies dominate that list. Thus, there is a need for more dialogue within the community on HRFv. We argue that HRFv causes a measurable impact on the BOLD time series, which, if ignored, will confound fMRI outcomes such as connectivity. We substantiate our argument with prior human HRFv research, including those focusing on rs-fMRI connectivity. We also present limited new data (to substantiate our ‘perspective’) on HRFv across two important demographic variables: age and sex. Lastly, to cover structures of the central nervous system beyond the brain, we also discuss the implications of HRFv for spinal cord fMRI.

## 2. The problem of HRF variability

HRFv was first demonstrated in 1998 (Aguirre et al., 1998) and further examined in later years (Miezin et al., 2000; Handwerker et al., 2004; de Zwart et al., 2005; Badillo et al., 2013). The HRF of a given brain region was identified to be different across individuals, and within a given individual, it was different across brain regions. Non-neural factors affecting the HRF is not merely the concern because a variable known HRF could be accounted for. Instead, the concern is that we do not understand these variable factors (and thus the HRF). The issue is alleviated in task fMRI studies by often modeling the HRF confound as time/dispersion derivatives of the canonical HRF in a general linear model (GLM) framework, which works well since BOLD is time-locked to an external stimulus. However, the outlook is different in rs-fMRI studies, which mostly ignore HRFv. The notion of HRF not being variable enough to be a serious confound was challenging to test, if not impossible, a few years ago because of the inability to estimate the HRF from rs-fMRI data. Necessary technical advancements [e.g., point-process theory (Tagliazucchi et al., 2012; Power et al., 2015)] have now resulted in rs-fMRI deconvolution techniques that are capable of HRF estimation from resting-state fMRI data (Havlicek et al., 2011; Karahanoğlu et al., 2013; Wu et al., 2013; Havlicek et al., 2015). We take a closer look at these techniques next.

## 3. HRF estimation from resting-state fMRI data

HRF estimation can be straightforward with two known quantities (fMRI and neural activity) and one unknown (HRF). This is possible with simultaneous fMRI and invasive recordings [e.g., (David et al., 2008; Wang et al., 2017b)], which is hardly feasible in humans. Obtaining simultaneous rs-EEG/fMRI data and considering EEG as the neural input to deconvolve fMRI [using AFNI’s *3dDeconvolve* (Feige et al., 2017)] is problematic because generative fMRI models do not consider scalp EEG as properly representing BOLD-inducing neural activity (Logothetis et al., 2001). A viable alternative is estimating HRF latency from a hypercapnic challenge because breath-hold causes vasodilation and modulates cerebral blood flow (CBF) (Thomason et al., 2007; Hall et al., 2016; McDonough et al., 2019) independent of neural activity, allowing us to measure vascular latency. Chang et al. (Chang et al., 2008) utilized this to correct for vascular latency prior to connectivity analysis. The disadvantage is that an additional breath-hold scan is not always feasible or available. Breath-hold is prone to subjective performance and can be challenging in those with some neurological diseases (Spano et al., 2013; Urback et al., 2017). Moreover, it only measures one aspect of HRF shape (TTP), while the entire HRF impacts BOLD.

To circumvent these concerns, an alternative is to perform blind deconvolution; that is, solve the mathematically ill-posed inverse problem of having two unknowns (HRF and neural activity) and one known (fMRI). This is feasible because the natural limits and biophysics of HRF and fMRI are well understood. HRF estimation then becomes a constrained optimization exercise. Such deconvolution techniques primarily focusing on task fMRI data (Gitelman et al., 2003; Gaudes et al., 2011; Hernandez-Garcia and Ulfarsson, 2011; Khalidov et al., 2011; Lopes et al., 2012; Bush and Cisler, 2013; Caballero Gaudes et al., 2013; Bush et al., 2015) are generally robust to HRF misspecification within a narrow physiological range, but are not viable for estimating voxel-specific HRFs in the entire brain, especially in rs-fMRI data. Whole-brain HRF estimation is preferable with rs-fMRI data because, with task fMRI, a given task does not activate the entire brain uniformly (Taylor et al., 2018), and even among activated voxels, the BOLD response is mostly non-uniform (Gonzalez-Castillo et al., 2012), sometimes leading to biologically implausible HRF estimates (Taylor et al., 2018). Some techniques that might, in principle, be viable for rs-fMRI have never been tested using rs-fMRI data (Sreenivasan et al., 2015; Cherkaoui et al., 2019).

This leaves us with four rs-fMRI deconvolution techniques that have been more widely adopted: (i) Wu et al.’s (2013) data-driven rsHRF method (Wu et al., 2021), (ii) parametric generative state-space models proposed within the stochastic dynamic causal modeling (DCM) framework (Havlicek et al., 2011; Friston et al., 2013, 2014), (iii) physiologically informed DCM (Havlicek et al., 2015; developed for task fMRI but can be extended to rs-fMRI), and (iv) Total Activation (Karahanoğlu et al., 2013; estimates an “activity-inducing signal” from BOLD, from which it is possible in principle to estimate the HRF by Wiener deconvolution), which has been applied in the brain (Karahanoğlu and Van De Ville, 2015; Zöller et al., 2021) as well as the spinal cord (Kinany et al., 2020). Notably, a substantial number of studies examining HRFv (described later) utilized Wu et al.’s technique (Wu et al., 2013), hence we describe it briefly here. The Wu et al. technique models rs-fMRI data as event-related time series, with events modeled as point-processes (Saad et al., 2012). Then it estimates the best-fit HRF in a least-squares sense, using the BOLD time series at identified events. Finally, the latent neural time series is estimated using Wiener deconvolution from the measured BOLD and the estimated HRF (Glover, 1999). This technique has been validated using simulations, non-invasive and invasive data (Tagliazucchi et al., 2012; Wu et al., 2013; Rangaprakash et al., 2018b; Wu et al., 2021), and has been applied in many recent papers (Amico et al., 2014; Lamichhane et al., 2014; Boly et al., 2015; Rangaprakash et al., 2017a,b,c, 2018a,b). HRFs separated by 4 weeks demonstrated moderate test–retest reliability (ICC = 0.51; Rangaprakash et al., 2020), which is impressive by current neuroimaging standards (Noble et al., 2019). A vast majority of human rs-fMRI studies do not perform additional breath-hold scans or do not have invasive recordings. This article focuses on the HRF confound in such studies, although some of the material is applicable generally to all fMRI studies. We next present the impact of HRFv on rs-fMRI data.

## 4. The confound of HRF variability on connectivity estimates

We focus here on functional connectivity (FC; Power et al., 2011) because most rs-fMRI studies investigate FC, or metrics derived from FC such as dynamic connectivity and graph measures. It is, however, notable that the HRF confound has also been investigated for effective connectivity models such as DCM (Friston et al., 2013), Granger causality (Deshpande et al., 2010; Lacey et al., 2014; Feng et al., 2015; Sreenivasan et al., 2015; Hampstead et al., 2016), and multivariate dynamical system models (Ryali et al., 2011, 2016a,b). Effective connectivity estimated from fMRI data is viable (Deshpande et al., 2010; Handwerker et al., 2012) as well as accurate (David et al., 2008; Havlicek et al., 2010, 2011; Wang et al., 2017a) only after deconvolution.

FC studies have, however, largely ignored HRFv either with the assumption that the HRF is similar enough among brain regions and individuals or due to the unavailability of HRF estimation methods. Recent evidence suggests that these assumptions must be re-evaluated. Although researchers have been aware, since the early days of fMRI, that BOLD measures blood oxygenation and not neural activity, the magnitude of HRF confound on FC is being investigated only recently with the availability of deconvolution techniques. We take a closer look at these. It has been demonstrated that the HRF is variable (Aguirre et al., 1998; Handwerker et al., 2004; Tak et al., 2015; Rangaprakash et al., 2018b; Bright et al., 2020) and recent reports suggest that ignoring it can introduce confounds in FC estimates (Rangaprakash et al., 2017b,c, 2018b; Yan et al., 2017, 2018). In fact, our study reported significant HRFv in the brain’s default mode network (DMN) that confounded FC by about 14.7% (Rangaprakash et al., 2018b). This error in FC estimates due to HRFv (FC-error) was smaller for within-lobe FC (12.6%) vs. between-lobes (15.6%), perhaps due to more variable vasculature in the latter case.

The HRF is not only different across individuals, but also different across clinical populations due to impairments in various factors contributing to HRFv. Our prior work has highlighted this in autism (Yan et al., 2018), post-traumatic stress disorder (PTSD) (Rangaprakash et al., 2017b), obsessive–compulsive disorder (OCD) (Rangaprakash et al., 2020), bipolar disorder and schizophrenia (Yan et al., 2022). They also demonstrated that HRF impairments are significant enough to confound FC group differences; and sometimes the confound was of a similar order of magnitude as FC impairments in these diseases. Other labs have made similar observations. For example, another study found that HRF alterations confound FC (Archila-Meléndez et al., 2020). HRFv also confounded rat FC (Peng et al., 2019). Using variable HRFs that are person-specific (vs. fixed HRF) improved connectivity estimates (Duffy et al., 2021). Taken together, emerging evidence indicates that HRFv is concerning for rs-fMRI connectivity and clinical applications.

## 5. The importance of studying HRF variability

Non-invasive measurements are not as “clean” as invasive ones. There is always an effort to make fMRI data as “clean” as possible by maximizing relative variance from neural sources. Examples of such efforts include minimizing physiological/thermal noise through ultra-high-field imaging (7T or greater field strength; Barry et al., 2018), improved acquisition sequences (Barth et al., 2016; Islam et al., 2019), and better denoising (Glover et al., 2000; Power et al., 2015). Today we are in a reproducibility crisis in functional imaging (Gorgolewski and Poldrack, 2016; Poldrack et al., 2017; Osmanlıoğlu et al., 2020), indicating that further advancements are needed to make this technology clinically more useful. We still do not understand the substantial intra- and inter-subject variability of fMRI outcomes. Further characterization of this variability is timely to maximize the percentage variance in BOLD explained by the underlying neural activity.

Effective modeling of HRFv could potentially contribute to this effort. Like head motion or physiological noise, HRFv is an undesirable confound reducing fMRI data fidelity. We predict that minimizing HRFv in fMRI data will improve data quality and enhance clinical discovery. Concerns about the HRF confound also exists among the broader neuroscience community. Examples include the viewpoint of cellular neuroscientists (Hall et al., 2016), special issue articles on HRFv (Ekstrom, 2021), studies on non-neural factors and BOLD (Das et al., 2021), and investigating the link between fMRI and neural activity (Mishra et al., 2021).

## 6. Clinical research and HRF variability

Evidence suggests that HRFv is relevant for clinical and geriatric research. HRFs in older adults are different from their younger counterparts. Older adults have longer TTP and shorter RH, largely due to vascular factors (West et al., 2019). Such change is associated with Alzheimer’s disease as well (Shan et al., 2016). Longer TTP is also linked to reduced intelligence (Anderson et al., 2020). Aberrant HRFs have been observed in stress (Elbau et al., 2018), mild traumatic brain injury (Mayer et al., 2014), aging (West et al., 2019; Yabluchanskiy et al., 2020; Tsvetanov et al., 2021), isolated cervical dystonia (Berman et al., 2020), and levels of consciousness (Gemma et al., 2009; Wu et al., 2019). Such HRF changes are concerning because they are at least partly driven by non-neural factors and can confound FC group differences. Although studying non-neural factors in brain disorders is a valid enterprise, attributing FC group differences entirely to neural activity is problematic. HRFv in neurological disorders is yet to be investigated; however, our prior work in psychiatric conditions [autism (Yan et al., 2018), PTSD (Rangaprakash et al., 2017b), schizophrenia and bipolar disorder (Yan et al., 2022)] demonstrated that HRF impairments in these conditions invariably confounds FC group differences, and the confound can sometimes be of the same order of magnitude as group differences. Taken together, HRFv has implications across a spectrum of cognitive, psychiatric and geriatric domains, and perhaps also in other cases in which HRFv has yet to be investigated.

## 7. Demographic variables and HRF variability

Non-neural factors that affect the HRF also differ across two highly relevant variables – age and sex. Aging causes vascular degradation; blood vessels of older adults are stiffer and less pulsatile, typically resulting in weaker BOLD responses to the same magnitude of neural activity (i.e., shorter RH) as well as longer time for peak BOLD activity (longer TTP; West et al., 2019; Yabluchanskiy et al., 2020; Tsvetanov et al., 2021). There is evidence for altered HRF in older adults (West et al., 2019; Tsvetanov et al., 2021), although the confound of HRFv on young vs. old FC group differences has not yet been studied. There is motivation to hypothesize that accounting for HRFv is essential for minimizing these vascular and other non-neural age-related confounds in rs-fMRI data. In fact, a recent report (Tsvetanov et al., 2021) noted that “*vascular confounds in fMRI studies are common. Despite over 10,000 BOLD-fMRI papers on aging, fewer than 20 have applied techniques to correct for vascular effects*.”

Factors affecting the HRF also differ between women and men. Men have lower CBF independent of neural activity (Ibaraki et al., 2010; Aanerud et al., 2017), which affects the HRF (Golestani et al., 2016; Kim and Ress, 2016). Other factors that exhibit sex differences include vascular physiology (Boese et al., 2017), capillary microcirculation (Huxley and Kemp, 2018), and overall cerebral hemodynamics (Barnes, 2017). Ignoring these factors by assuming a fixed canonical HRF is a potential confound in rs-fMRI studies that report data from both sexes, and/or perform women vs. men group comparisons. Despite this, HRF sex differences have never been directly studied. Hence, we next present limited data in this context.

## 8. Results on HRF differences between sexes and their impact on connectivity

We utilized 7 T rs-fMRI data from our earlier HRF study [*N* = 47, 22F/25M, healthy adults; Rangaprakash et al., 2018b; data made public (Rangaprakash et al., 2018c)] (please refer to these publications for data details). Kindly note that since this is a perspective article, we have not presented comprehensive results, but we hope that the results herein will encourage extensive follow-up studies. Briefly, upon standard pre-processing, we extracted mean region-of-interest (ROI) time series from DMN regions defined by the Power-Petersen atlas (Power et al., 2011) and estimated FC between all DMN ROI pairs using Pearson’s correlation (Mejia et al., 2018; Noble et al., 2019). This procedure was repeated for two separate pipelines: data with deconvolution (DC) and no deconvolution (NDC), which differed only in HRFv. We also obtained HRF parameters for each ROI during deconvolution.

**Figure 2:**
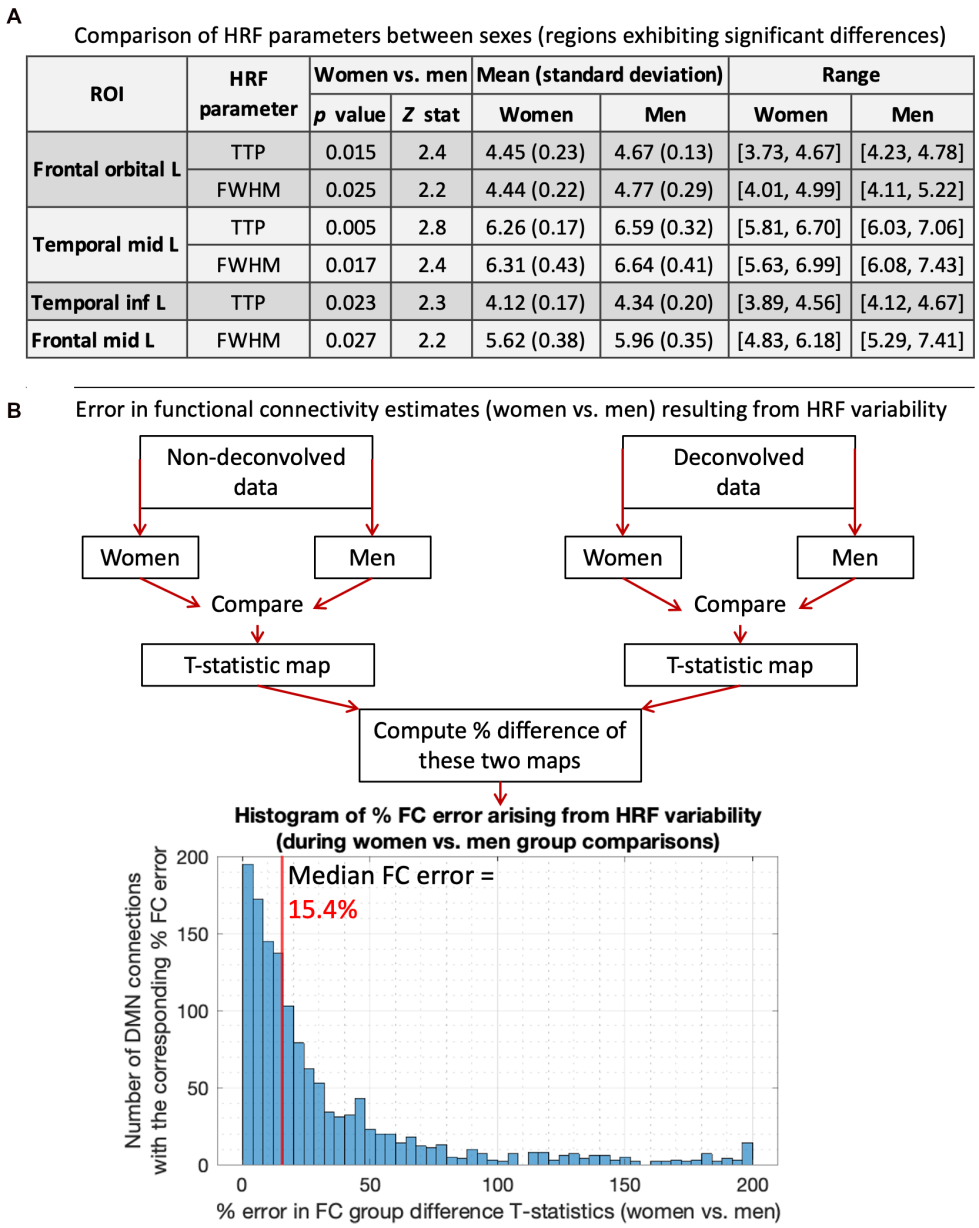
HRF results for women vs. men. **(A)** Comparing HRF parameters between men and women, showing the four regions that exhibited significant HRF time-to-peak (TTP) and/or full-width-at-half-max (FWHM) differences. TTP and/or FWHM were significantly higher in men. The 5th and 95th percentile values were used for range. **(B)** A flowchart illustrating our methodology to compute errors in FC estimates arising from HRF variability, and the results presented below that. The histogram shows the distribution of % FC error arising from men vs. women HRF differences. The median FC error was 15.4%, with a 95% confidence interval of [1.5–131.6%].

We compared ROI-level HRF parameters between men and women (*p* < 0.05, FDR corrected). T-test was used for RH, and Wilcoxon rank-sum test was used for discrete variables (TTP, FWHM). There were no RH differences, but men had significantly longer TTP and/or FWHM in four regions (Figure 2A). Longer TTP/FWHM in men could be due to CBF and vascular differences between sexes (Ibaraki et al., 2010; Golestani et al., 2016; Aanerud et al., 2017; Barnes, 2017; Boese et al., 2017; Huxley and Kemp, 2018). The mean values averaged across all ROIs and subjects were as follows: RH (women = 5.01, men = 4.77), TTP (women = 5.61 s, men = 5.73 s), FWHM (women = 5.62 s, men = 5.79 s).

Next, we computed the error in FC group difference T-stats resulting from HRF differences. For this, women vs. men FC stats were performed separately for DC and NDC pipelines, and the percentage difference in resulting statistical maps was computed (Figure 2B). HRF differences between sexes resulted in a 15.4% median FC error. After thresholding (*p* < 0.05, FDR corrected), all three identified FCs were false positives (FC of temporal_inf_L/frontal_sup_L/fusiform_L with temporal_mid_L). Although only four regions and three connections exhibited significant HRF and FC differences, respectively, it must not be inferred that the rest of the regions or connections were unaffected by HRFv. These HRF/FC differences were large enough to be detected for our sample size and data quality, but that does not mean that the difference can be ignored in the other connections. The testament to this fact is that HRF differences between sexes resulted in 15.4% median FC error with contribution even from a vast number of regions/FCs outside of the significant ones (Figure 2). Our prior work has demonstrated the same (Rangaprakash et al., 2017b, 2018b; Yan et al., 2018, 2022). Taken together, a measurable portion of sex differences in DMN were attributable to HRFv.

As a secondary result not related to sex differences, we also report whole-brain-level average metrics of variability in HRF parameters within and between healthy young adult subjects, because these were not reported in our earlier publication (Rangaprakash et al., 2018b), but they support our conclusions and encourage future work. Of course, for the nature of this article, these results could be more complex and extensive. Using the same FC data, we (i) computed within-subjects variability of each HRF parameter as the mean percentage difference between all ROI pairs; and (ii) computed between-subjects variability of each HRF parameter as the mean percentage difference between all pairs of subjects. We used a *t*-test for RH and a rank-sum test for TTP/FWHM (*p* < 0.05, FDR corrected). Within-subjects, we found on average 14.1% RH variability, 13.5% TTP and 13.4% FWHM variability. L/R orbitofrontal had the largest HRF difference with other regions (86%/65% RH, 43%/24% TTP, 42%/24% FWHM variability), perhaps due to susceptibility (Buxton, 2010) and/or vascular (Tsvetanov et al., 2021) differences. Temporal lobe had larger variability than other lobes in RH (*p* = 0.034), TTP (*p* = 0.039) and FWHM (*p* = 0.037). Between-lobe variability was higher than within-lobe in RH (*p* = 0.039), TTP (*p* = 0.028) and FWHM (*p* = 0.041) (possibly due to vastly different vasculature across lobes). Between-subjects HRFv (RH: 29.8%, TTP: 28.5%, FWHM: 28.1%) was larger than within-subjects. The occipital lobe varied significantly less across subjects than other lobes in RH (*p* = 0.048) and TTP (*p* = 0.045).

## 9. The HRF in the spinal cord

The central nervous system (CNS), which includes the brain and the spinal cord, is a single continuous entity. But prior HRF literature is exclusively focused on the brain. Spinal cord rs-fMRI studies have so far not accounted for HRFv, except for a recent study (Kinany et al., 2020). If systematic brain HRF changes in pathological conditions translate to the cord, it is concerning because the cord is clinically relevant for several neurological diseases [e.g., multiple sclerosis (Conrad et al., 2018), chronic pain (Reckziegel et al., 2019), amyotrophic lateral sclerosis (de Albuquerque et al., 2017), transverse myelitis (Cacciaguerra et al., 2019), ataxia (Faber et al., 2021), and spinal cord injury (Freund et al., 2019)]. Cord impairments are being discovered in other pathologies [Alzheimer’s disease (Lorenzi et al., 2020) and cerebral palsy (Trevarrow et al., 2021)], suggesting that more disorders could involve the cord than we currently understand. HRFv could confound cord FC impairments in these diseases as well. Hence, characterizing HRFv in the spinal cord is clinically relevant and novel.

## 10. Discussion and conclusions

Herein we described prior evidence for HRFv and its confound on rs-fMRI FC and elaborated on this research’s importance and clinical relevance. Unexplained variability in BOLD is a more significant concern today than before because connectivity is used in sophisticated contexts such as dynamics (Preti et al., 2016), single-subject-level prediction (Jollans et al., 2019), laminar fMRI (Finn et al., 2020), and precision medicine (Finn et al., 2015). HRFv matters to a larger extent for all the desired ‘precision’ and fidelity expected of rs-fMRI today. With fast fMRI acquisition becoming prevalent (Preibisch et al., 2015; Barth et al., 2016), accounting for HRFv is even more critical (Lewis et al., 2018) to determine the neural/vascular origin of fMRI timing differences. Thus far, FC error arising only from spatial HRFv has been quantified, and only in parts of the brain and in small samples (Rangaprakash et al., 2018b). We provided limited data for FC error between sexes. Further research is required to quantify HRFv and FC error across various within- and between-subject comparison scenarios and demographic variables.

Taken together, measurements of FC often involve unexplained variance between 40% (ML prediction) and 70% (behavioral association; Finn et al., 2015; Jollans et al., 2019). While the underlying prediction/association models may be statistically significant and perform above chance, there is still a sizeable unexplained variance. We, therefore, argue that the HRF confound, typically in the range of 10–30%, may explain a part of this variance. We thus conclude that HRFv cannot be ignored in rs-fMRI studies, and it should be commonly accounted for during rs-fMRI data pre-processing.

## Data availability statement

The datasets presented in this study can be found in online repositories. The names of the repository/repositories and accession number(s) can be found at: doi.org/10.1016/j.dib.2018.01.003.

## Ethics statement

The studies involving human participants were reviewed and approved by Auburn University Institutional Review Board. The patients/participants provided their written informed consent to participate in this study.

## Author contributions

DR: conceptualization, methodology, software, data analysis, investigation, visualization, writing–original draft, and reviewing and editing. RB: investigation, writing–reviewing and editing, and supervision. GD: conceptualization, methodology, data acquisition, investigation, writing–reviewing and editing, and supervision. All authors contributed to the article and approved the submitted version.

## Funding

This work was supported by National Institutes of Health (NIH) through grants R01EB027779 and R21EB031211 (RB) and R01EY025978 (GD). Support was also received through the Athinoula A. Martinos Center for Biomedical Imaging (RB) and the Auburn University MRI Research Center (GD). The content is solely the responsibility of the authors and does not necessarily represent the official views of the NIH.

## Conflict of interest

The authors declare that the research was conducted in the absence of any commercial or financial relationships that could be construed as a potential conflict of interest.

## Publisher’s note

All claims expressed in this article are solely those of the authors and do not necessarily represent those of their affiliated organizations, or those of the publisher, the editors and the reviewers. Any product that may be evaluated in this article, or claim that may be made by its manufacturer, is not guaranteed or endorsed by the publisher.
